# How Ethical Is Our Current Delivery of Care to Patients with Severe and Complicated Obesity?

**DOI:** 10.1007/s11695-018-3301-1

**Published:** 2018-05-15

**Authors:** Hilary Craig, Carel le Roux, Fiona Keogh, Francis M. Finucane

**Affiliations:** 1 Department of Surgery, St. James’s Hospital, Dublin, Ireland; 2 Diabetes Complications Research Centre, Conway Institute, University College Dublin, Dublin, Ireland; 3 Centre for Economic and Social Research in Dementia, National University of Ireland, Galway, Ireland; 4 Bariatric Medicine Service and HRB Clinical Research Facility, Galway University Hospital and National University of Ireland, Galway, Ireland

**Keywords:** Justice, Beneficence, Non-maleficence, Autonomy, Bariatric, Ethics

## Abstract

Despite overwhelming evidence that bariatric interventions reduce morbidity and mortality and are cost-effective, access for affected patients is limited. We sought to describe the extent to which health policy makers and publically funded health services have an ethical obligation to provide bariatric care. We conducted a narrative review of the literature pertaining to the efficacy, safety, and cost-effectiveness of bariatric surgical interventions, in the context of the core principles of medical ethics. We found that in relation to autonomy (i.e., the right to self-determination), beneficence, non-maleficence, and justice (i.e., the obligation to provide fair and equitable treatment to all patients), the current provision of bariatric surgical care fell short of meeting internationally recognized medical ethical standards. These findings have important implications for government policy and healthcare resource allocation. Respecting the individual’s right of self-determination, to do good, prevent harm, and provide equity in access to services is paramount, even when that individual is obese.

## Introduction

While the global prevalence of obesity is increasing [1], the rise in the number of patients with severe obesity (body mass index (BMI) > 40 kg/m^2^) has been particularly dramatic [2]. Simultaneously, evidence has accumulated that bariatric surgery is efficacious [3] and cost-effective [4]. Although approximately 80% of patients with severe obesity are medically and psychologically suitable for bariatric surgery, only 10% want to pursue this option [5]. Potentially eligible patients may decline bariatric surgery, or may be reluctant to be referred to specialist bariatric services because of previous negative interactions with health professionals, low self-esteem, or embarrassment [6]. Recent estimates from a nationally representative cohort study of adults over 50 years old (TILDA, the Irish Longitudinal Study on Ageing) suggest the prevalence of eligibility for bariatric surgery in older adults with a BMI ≥ 35 kg m^−2^ and one or more comorbidities such as type 2 diabetes (T2D) or sleep apnea was 7.4% [7]. Despite the clearly defined efficacy, cost-effectiveness, and need for bariatric surgery, often less than 0.1% of eligible patients receive publicly funded procedures internationally. For example, in Ireland, fewer than 1 per 100,000 people receive surgery, in contrast to bariatric surgery rates of 70 per 100,000 in Sweden and France and 50 per 100,000 in the USA [8], despite these countries having the same European laws. In addition to international variations based on different policies and health systems in different countries, socioeconomic factors play a major role in determining which eligible patients receive bariatric surgery [9]. Adolescents frequently encounter obstacles to treatment authorization from insurance carriers, with only approximately half of eligible patients being approved by insurers, despite fulfilling criteria [10]. Such delays and prevarication are harmful to patients. In one US study, delayed access to surgery was associated with a three-fold increased mortality over a decade compared to timely provision of surgery in patients who wanted and needed it [11].

While some European governments have recognized obesity as a disease since 2005 and committed to develop bariatric surgical services [12], programmatic funding is often lacking. Inadequate access to bariatric care is a source of concern and frustration for health professionals and patients. Much of the resultant discourse on the topic is subjective and emotive and has failed to influence policy. We sought to explore objectively the extent to which the failure to provide bariatric surgical care breaches the core principles of medical ethics and the human rights of affected individuals.

Medical ethical principles can be conceptualized in different ways. Immanuel Kant, one of the foremost philosophers of the eighteenth century, described deontology, the “science of duty,” as a consideration of the righteousness of any action rather than its consequences in determining how morally and ethically sound it was [13]. Conversely, consequentialism is a class of normative ethical theories holding that the consequences of ones conduct or actions are the ultimate basis for any judgment about the rightness or wrongness of that conduct [14]. There is also the concept of virtue ethics, which emphasize the virtue of moral character [15]. Utilitarianism focusses on the common good [16]. So, a deontologist would provide care to a patient because it is the right thing to do, a consequentialist would see that it would end up being good for the patient, the virtuist would do it to reflect their moral integrity, and the utilitarian would see the collective good. While each of these concepts has its merits, they were largely superseded in 1979 when Beauchamp and Childers described principlism in their textbook on the principles of biomedical ethics [17]. These principles are autonomy, beneficence, non-maleficence, and justice and are viewed by many as the standard theoretical framework from which to analyze ethical considerations in medicine. The principles are debated (for example, utilitarianism becomes more relevant in confronting acute severe epidemics), but they do resonate with social moral norms and will form the basis for our consideration of how inadequate provision of bariatric surgical care is ethically unsound.

## Autonomy

Autonomy refers to an individual’s right to self-determination and their ability to make decisions about their medical treatment based on their own beliefs and values. It is the foundation for informed consent. There are three criteria that define a state of autonomy: intentionality, understanding, and non-control, i.e., an absence of undue influence or coercion. While autonomy has only become an important ethical consideration in the last few decades [17], it is now legally endorsed in most jurisdictions, such as in the European Convention on Human Rights Act (2003). Arguably, the appalling lapses in medical ethical standards that were apparent in the Tuskegee Syphilis Study [18, 19] are being replicated in our treatment of obese patients, 50 years later. The failure to provide access to efficacious, cost-effective bariatric surgical interventions and the constraint of treatment choice constitutes a breach of patient autonomy. While we know that obesity is a complex disease [20] and the archaic, pejorative view that it represents a lack of discipline and self-control in affected individuals [21] has largely been dismissed, stigma still affects obese patients in a number of ways. Social media discourse about obesity tends to be derogatory and misogynistic [22]. Doctors are as biased as the general public against people with obesity [23] and 24% of nurses in one study described feeling “repulsed” by patients who were obese [24]. These strongly negative stereotypes held by some healthcare professionals influence their judgment, interpersonal behavior, and decision-making around patient care [25]. Experiences and expectations of poor treatment may lead to distress and avoidance of care, mistrust of doctors, and poor adherence among affected patients. Half of patients with obesity have weight bias internalization, or self-stigmatization [26] and this is known to hinder weight loss maintenance in adults undergoing therapeutic interventions for obesity [27]. Where stigma and bias in healthcare limit access to proven, efficacious interventions, it constitutes an unacceptable breach of patients’ right to autonomy.

## Beneficence

There is overwhelming evidence now that bariatric surgery is efficacious and cost-effective. A Cochrane review of 22 randomized controlled trials found bariatric surgery was more clinically effective and cost-effective for treating severe obesity than medications or lifestyle modification, after 2 years [28]. Trials with longer follow-up also favor surgery [29]. The Swedish Obese Subject study of over 4000 patients who underwent either surgery or lifestyle modification has shown more than 15% weight loss being maintained for 20 years after surgery, with improvements in comorbidities such as diabetes [30]. Swedish registry data show a 58% reduction in mortality after 4 years’ follow-up of 6000 patients with diabetes who underwent bariatric surgery, compared to matched patients without surgery [31]. Typical reported weight loss after gastric bypass is 15–25% after 20 years for severely obese patients [30], compared to 7% mean weight loss with intensive lifestyle modification programs or novel medical therapies [32, 33].

Aside from the compelling evidence that bariatric surgery is efficacious, there is also little doubt about its cost-effectiveness. Several health technology assessments (HTA) have found that for patients with a body mass index (BMI) ≥ 40 kg m^−2^, the incremental cost-effectiveness ratios (ICER) for surgery ranged between US$ 2700 and US$ 5400 per quality-adjusted life year (QALY) gained over 20 years [34], well below the QALY threshold for cost-effectiveness used in most jurisdictions. For patients with diabetes and a BMI of 30–39 kg m^−2^, the ICER fell to US$ 1900 per QALY [34]. These figures are in line with those for other public health interventions such as statins for primary prevention of cardiovascular disease [35]. Economic analyses confirm that the investment required for surgery seems justified [36]. In patients with diabetes, for example, the cost of surgery will be recouped within 3 years through reduced direct healthcare costs driven by fewer prescriptions [37]. Modeled data also suggest that surgery has indirect cost benefits, such as reduced state disability allowances if improved activity levels allow patients to return to paid employment after surgery [38]. There is no doubt, then that the provision of bariatric surgical care to appropriately selected patients “does good” and fulfills the ethical principle of beneficence.

## Non-maleficence

Adhering to the principle of “primum non nocere” (first do no harm) in reality involves trying to ensure that the potential benefits of an intervention outweigh the potential harms. The consideration of ethical aspects of harm goes beyond potential complications from bariatric surgery. John Stuart Mill, a proponent of utilitarianism, developed the so-called harm principle to inform thresholds for acceptable harm and in particular the extent to which legislation can and should be enacted to mitigate any perceived harm for public health [39]. Essentially, where individuals risk harm only to themselves through specific actions, there is less of an imperative to restrict personal freedom than if those actions pose risks to others. Thus, legislation to mitigate the effects of exposure to passive smoking or excessive vehicular speed would be warranted.

Recently, exposing obese individuals to potential harm on the grounds that their excess body weight imposes costs on others has been espoused in mainstream, contemporary bioethical discourse. For example, Peter Singer, a prominent ethics scholar, has argued that heavier airline passengers should be charged more to fly [40]. He referred to data from the Australian airline Quantas, where an increase in the weight of the average passenger of 2 kg from 2000 was noted, costing an extra $472 per flight in fuel between Sydney and London, or one million dollars per annum. He argued that such harms and costs caused by obese people justified public policies “that discouraged weight gain.” Dan Callahan, another leading bioethicist, went further in proposing coercive public health measures to try to induce overweight people to lose weight [41]. This focus on making obese people “aware” of their lack of “moral fiber” and “self-control” is pervasive in popular media [22], leading to self-stigma, which aside from diminishing autonomy as noted above, also causes harm [25]. This culture of acceptance of harm to obese individuals pervades aspects of public health policy, such as implementation of childhood obesity screening programs which have potential to harm, without evidence that they do much good [42].

However, Christopher Mayes countered that Singer’s and Callahan’s arguments are flawed because their invocation of the harm principle relies on “superficial readings of public health research to amplify the harm caused by obese individuals and ignore pertinent epidemiological research on the social determinants of obesity” [43]. He argues that structural and environmental factors rather than individual choice should be the focus for obesity prevention strategies. Otherwise, we risk “harming the already harmed.” While these considerations apply to people with obesity in general, the reliance on coercive public health measures that magnify stigma and harm may also perpetuate a pejorative, moralistic view of severely obese patients that diminishes the political imperative to provide them with the bariatric healthcare that they need.

## Justice

The final principle of medical ethics is justice, which reflects the need for a fair and equal distribution and allocation of healthcare resources in society. There are three domains to the concept of ethical justice. Firstly, there is recognition that all resources are finite. Secondly, the rights of individuals need to be respected and thirdly, morally established and accepted laws need to be upheld [17]. There needs to be a process, therefore, of rationing and triage so that the deployment of finite resources can be prioritized. The most conventional and rational approach is to consider the clinical efficacy of an intervention relative to its costs as well as its potential harms. This is the basis for contemporary health economics and measures of cost-effectiveness and cost utility such as the cost per quality-adjusted life year (QALY). We saw above that bariatric surgery is cost-effective [4] and indeed is cost saving in patients with T2D [44]. Suggestions that inadequate resources are the reason for the failure to provide bariatric surgical services to severely obese adults do not stand up to scrutiny: A large HTA in the UK found that if a decision maker was willing to pay US$ 27,000 for an additional QALY, the probability of bariatric surgery being cost-effective over 20 years would be 100% [34]. From a societal health equality perspective, given that health is essential for flourishing, there is no ethically sound rationale to encourage some to flourish more than others, and so all avoidable health inequalities should be minimized [45].

It seems that from a political perspective, adverse lifestyle choices forfeit the right to healthcare. This is reminiscent of the approach taken to patients with AIDS in the 1980s, where an emphasis on “ethical behavior” was preferred to prioritizing treatment for those who developed the disease. In the USA, recent amendments to the American Disability Act (ADA) [46] and the establishment of the Equal Employment Opportunity Commission have provided some protection for severely obese people from discrimination based on actual or perceived disability in the employment context. The American Disability Act defines disability as “a physical or mental impairment that substantially limits one or more of the major life activities of a person.” However, Michigan is the only US state with a law prohibiting discrimination against overweight people (the Elliott-Larsen Civil Rights Act) while other states such as Washington, California, Wisconsin, and Illinois have municipal codes against discrimination on the basis of height, weight, or physical characteristics. While there is currently no European legislation covering discrimination against obese people, the European Court of Justice (CJEU), using directive 2000/78 in a Danish unfair dismissal case, has ruled that it is illegal to discriminate against employees with obesity where their obesity constitutes a disability. In international law, article 12 of the International Covenant on Economic, Social and Cultural Rights (ICESCR) outlines obligations of nations to respect, protect, and fulfill their citizens’ right to health. Given that discrimination against individuals with obesity is real but also illegal and that obesity invariably constitutes a disability for bariatric patients, the current approach of health policy makers to deny the provision of bariatric service not only constitutes a breach of ethical justice but would also seem unwise.

## Conclusions

Clearly, ethical obligations go beyond the need for healthcare systems to invest in and provide bariatric care. For example, there is an ethical responsibility on clinicians to target treatment at bariatric patients with the greatest potential for improved health. Patients with obesity-related diseases that are expensive to treat and who are likely to need fewer medications afterwards, such as those with recently diagnosed type 2 diabetes, are an obvious priority [36]. Alternatively, those with established microvascular complications of diabetes might be a good group to prioritize, because even with optimal drug therapy, many will become increasingly unwell from those complications and require more expensive treatment over time [47]. Moreover, there are ethical obligations on patients themselves, to engage meaningfully in longer term follow-up strategies and to see bariatric surgery not as a stand-alone intervention, but rather as part of a broader process of bariatric care.

Notwithstanding these considerations, the current lack of provision of bariatric surgical care to patients in some jurisdictions constitutes a breach of each of the four principles of contemporary biomedical ethics. Because patients have a right to access this treatment, they stand to benefit from it. Reliance on other strategies such as coercive public health measures may be ineffective or even harmful and because it is cost-effective and represents good value for money. However, pervasive bias and stigma against people with obesity perpetuates political inertia to provide these services and facilitates increasingly ethically unsound neglect of this vulnerable patient group [48]. These observations have important implications for government policy, for healthcare resource allocation, and for future clinical programs for obesity treatment. Respecting the individual’s right of self-determination, doing good, preventing harm, and allocating resources fairly and transparently should be the objective for all health policy makers, even when considering the care of individuals with severe obesity.

## References

[1] Trends in adult body-mass index in 200 countries from 1975 to 2014: a pooled analysis of 1698 population-based measurement studies with 19.2 million participants. The Lancet. 2016;387(10026):1377–96. 10.1016/S0140-6736(16)30054-X PMC7615134 27115820

[2] Sturm R, Hattori A (2013). Morbid obesity rates continue to rise rapidly in the United States. Int J Obes.

[3] Reges O, Greenland P, Dicker D, Leibowitz M, Hoshen M, Gofer I (2018). Association of bariatric surgery using laparoscopic banding, Roux-en-Y gastric bypass, or laparoscopic sleeve gastrectomy vs usual care obesity management with all-cause mortality. JAMA.

[4] James R, Salton RI, Byrnes JM, Scuffham PA (2017). Cost-utility analysis for bariatric surgery compared with usual care for the treatment of obesity in Australia. Surg Obes Relat Dis.

[5] Jennings A, Hughes CA, Kumaravel B, Bachmann MO, Steel N, Capehorn M (2014). Evaluation of a multidisciplinary tier 3 weight management service for adults with morbid obesity, or obesity and comorbidities, based in primary care. Clin Obes.

[6] Owen-Smith A, Donovan J, Coast J (2014). “Vicious circles”: the development of morbid obesity. Qual Health Res.

[7] O’Neill KN, Finucane FM, le Roux CW, Fitzgerald AP, Kearney PM. Unmet need for bariatric surgery. Surgery for Obesity and Related Diseases. 2017;13(6):1052–6. 10.1016/j.soard.2016.12.015 28256395

[8] Angrisani L, Santonicola A, Iovino P, Formisano G, Buchwald H, Scopinaro N (2015). Bariatric surgery worldwide 2013. Obes Surg.

[9] Martin M, Beekley A, Kjorstad R, Sebesta J (2010). Socioeconomic disparities in eligibility and access to bariatric surgery: a national population-based analysis. Surg Obes Relat Dis.

[10] Inge TH, Boyce TW, Lee M, Kollar L, Jenkins TM, Brandt ML (2014). Access to care for adolescents seeking weight loss surgery. Obesity.

[11] Flanagan E, Ghaderi I, Overby DW, Farrell TM (2016). Reduced survival in bariatric surgery candidates delayed or denied by lack of insurance approval. Am Surg.

[12] Polcwiartek C, Vang T, Bruhn CH, Hashemi N, Rosenzweig M, Nielsen J (2016). Diabetic ketoacidosis in patients exposed to antipsychotics: a systematic literature review and analysis of Danish adverse drug event reports. Psychopharmacology.

[13] Kant I. Critique of pure reason. Cambridge: Cambridge University Press. 1998. p. 785.

[14] Roache R (2015). Making consequentialism more appealing. J Med Ethics.

[15] Gardiner P (2003). A virtue ethics approach to moral dilemmas in medicine. J Med Ethics.

[16] Gillon R (1985). Utilitarianism. Br Med J (Clin Res Ed).

[17] Beauchamp TL, Childress JF (2013). Principles of biomedical ethics.

[18] Thomas SB, Quinn SC (1991). The Tuskegee Syphilis Study, 1932 to 1972: implications for HIV education and AIDS risk education programs in the black community. Am J Public Health.

[19] Brandt AM (1978). Racism and research: the case of the Tuskegee Syphilis Study. Hast Cent Rep.

[20] O’Rahilly S, Farooqi IS (2008). Human obesity: a heritable neurobehavioral disorder that is highly sensitive to environmental conditions. Diabetes.

[21] Prentice AM, Jebb SA (1995). Obesity in Britain: gluttony or sloth?. BMJ : Br Med J.

[22] Chou W-yS, Prestin A, Kunath S (2014). Obesity in social media: a mixed methods analysis. Transl Behav Med.

[23] Sabin JA, Marini M, Nosek BA (2012). Implicit and explicit anti-fat bias among a large sample of medical doctors by BMI, race/ethnicity and gender. PLoS One.

[24] Puhl R, Brownell KD (2001). Bias, discrimination, and obesity. Obes Res.

[25] Phelan SM, Burgess DJ, Yeazel MW, Hellerstedt WL, Griffin JM, van Ryn M (2015). Impact of weight bias and stigma on quality of care and outcomes for patients with obesity. Obes Rev.

[26] Puhl RM, Himmelstein MS, Quinn DM (2018). Internalizing weight stigma: prevalence and sociodemographic considerations in US adults. Obesity.

[27] Puhl RM, Quinn DM, Weisz BM, Suh YJ (2017). The role of stigma in weight loss maintenance among U.S. adults. Ann Behav Med.

[28] Colquitt JL, Pickett K, Loveman E, Frampton GK. Surgery for weight loss in adults. Cochrane Database of Systematic Reviews. 2014;(8):243. 10.1002/14651858.CD003641.pub4 PMC9028049 25105982

[29] Courcoulas AP, Belle SH, Neiberg RH, Pierson SK, Eagleton JK, Kalarchian MA (2015). Three-year outcomes of bariatric surgery vs lifestyle intervention for type 2 diabetes mellitus treatment: a randomized clinical trial. JAMA Surg.

[30] Sjostrom L (2013). Review of the key results from the Swedish Obese Subjects (SOS) trial—a prospective controlled intervention study of bariatric surgery. J Intern Med.

[31] Eliasson B, Liakopoulos V, Franzen S, Naslund I, Svensson AM, Ottosson J (2015). Cardiovascular disease and mortality in patients with type 2 diabetes after bariatric surgery in Sweden: a nationwide, matched, observational cohort study. Lancet Diabetes Endocrinol.

[32] Pi-Sunyer X, Astrup A, Fujioka K, Greenway F, Halpern A, Krempf M (2015). A randomized, controlled trial of 3.0 mg of liraglutide in weight management. N Engl J Med.

[33] Wing RR, Bolin P, Brancati FL, Bray GA, Clark JM, Coday M (2013). Cardiovascular effects of intensive lifestyle intervention in type 2 diabetes. N Engl J Med.

[34] Picot J, Jones J, Colquitt JL (2009). The clinical effectiveness and cost-effectiveness of bariatric (weight loss) surgery for obesity: a systematic review and economic evaluation. Health Technol Assess.

[35] Marsh K, Dolan P, Kempster J, Lugon M (2013). Prioritizing investments in public health: a multi-criteria decision analysis. J Public Health (Oxf).

[36] Stegenga H, Haines A, Jones K, Wilding J (2014). Identification, assessment, and management of overweight and obesity: summary of updated NICE guidance. BMJ.

[37] Klein S, Ghosh A, Cremieux PY, Eapen S, McGavock TJ (2011). Economic impact of the clinical benefits of bariatric surgery in diabetes patients with BMI >/=35 kg/m(2). Obesity (Silver Spring).

[38] Hawkins SC, Osborne A, Finlay IG, Alagaratnam S, Edmond JR, Welbourn R (2007). Paid work increases and state benefit claims decrease after bariatric surgery. Obes Surg.

[39] Mill JS, Sher G (2001). Utilitarianism.

[40] Singer P. Weigh More, Pay More. Project syndicate . 2012. Available from https://www.projectsyndicate.org/commentary/weigh-more--pay-more.

[41] Callahan D (2013). Obesity: chasing an elusive epidemic. Hast Cent Rep.

[42] Geoghegan R, Kelly C, Finucane FM (2015). Should we screen for childhood obesity?. Clin Obes..

[43] Mayes C (2015). The harm of bioethics: a critique of singer and Callahan on obesity. Bioethics.

[44] Keating C, Neovius M, Sjoholm K, Peltonen M, Narbro K, Eriksson JK (2015). Health-care costs over 15 years after bariatric surgery for patients with different baseline glucose status: results from the Swedish Obese Subjects study. Lancet Diabetes Endocrinol.

[45] Culyer T (2015). Efficiency, equity and equality in health and health care.

[46] Pomeranz JL, Puhl RM (2013). New developments in the law for obesity discrimination protection. Obesity.

[47] Miras AD, Chuah LL, Khalil N, Nicotra A, Vusirikala A, Baqai N (2015). Type 2 diabetes mellitus and microvascular complications 1 year after Roux-en-Y gastric bypass: a case-control study. Diabetologia.

[48] Pearl RL, Wadden TA, Tronieri JS, Chao AM, Alamuddin N, Berkowitz RI. Everyday discrimination in a racially diverse sample of patients with obesity. Clinical Obesity. 2017;8(2):140–6. 10.1111/cob.12235 PMC5842103 29266824

